# The hsa_circ_0084050/miR-373-3p Axis Controls Bone Sialoprotein-Induced ADAM9 Upregulation to Drive Lung Cancer Progression and Metastasis

**DOI:** 10.3390/ijms27177840

**Published:** 2026-09-01

**Authors:** Le Huynh Hoai Thuong, Chang-Lun Huang, Chun-Lin Liu, Jeng-Hung Guo, Po-I Liu, Chih-Hsin Tang

**Affiliations:** 1 Department of Pharmacology, School of Medicine, China Medical University, Taichung 404, Taiwan; 2 Division of General Thoracic Surgery, Department of Surgery, Changhua Christian Hospital, Changhua 500, Taiwan; 3 Department of Neurosurgery, China Medical University Hospital, Taichung 404, Taiwan; 4 Department of Physical Therapy, Asia University, Taichung 404, Taiwan; 5 Department of General Thoracic Surgery, Asia University Hospital, Taichung 404, Taiwan; 6 Chinese Medicine Research Center, China Medical University, Taichung 404, Taiwan; 7 Office of Research & Development of Medical Laboratory Science and Biotechnology, Asia University, Taichung 404, Taiwan

**Keywords:** lung cancer, metastasis, proliferation, BSP, ADAM9

## Abstract

Lung cancer carries a high mortality burden, with metastatic dissemination accounting for much of its poor prognosis. Bone sialoprotein (BSP), a matricellular protein belonging to the SIBLING family, has been associated with tumor invasion; nonetheless, its role in lung cancer remains inadequately characterized. Clinical database analysis indicates that BSP is the most critical SIBLING protein associated with lung cancer proliferation and metastasis. Our clinical data also confirmed that BSP levels are higher in metastatic lung cancer compared to non-metastatic cases. We further demonstrate that BSP promotes lung cancer progression and motility through upregulated ADAM9 expression. Mechanistically, we revealed that BSP enhances ADAM9-dependent proliferation and motility via the FAK pathway. In addition, the hsa_circ_0084050/miR-373-3p regulatory axis contributes to BSP-mediated ADAM9 regulation. In an exploratory mouse metastasis model established by caudal artery injection of A549 control or stable BSP-shRNA cells into male BALB/c nude mice, BSP knockdown was associated with a lower metastatic tumor burden. Therefore, BSP may serve as a therapeutic target for limiting lung cancer progression and metastatic dissemination.

## 1. Introduction

Lung cancer continues to be the main cause of cancer-related deaths globally, mostly owing to its rapid advancement and often late-stage detection [1,2]. Although it accounts for only 12% of all new cancer cases, lung cancer is accountable for approximately 19% of worldwide cancer fatalities, underscoring its disproportionate lethality [3]. The high mortality rate is largely attributed to the disease’s strong metastatic potential, which involves complex molecular pathways enabling tumor cells to survive, migrate, and colonize distant sites [4]. Despite advances in molecular and immune-based therapies, survival outcomes remain poor, particularly because most cases are detected at advanced stages when curative options are limited [3,5]. Continued investigation into the molecular pathways of lung cancer is therefore crucial for the advancement of more efficient targeted therapies [6].

A disintegrin and metalloproteinase 9 (ADAM9) is an important mediator of tumor progression and has been observed to enhance invasion, proliferation, and migration in several malignancies, including lung cancer [7]. Mechanistically, ADAM9 facilitates metastasis by suppressing miR-1 and activating CDCP1 via EGFR signaling, as well as by enhancing brain colonization through tissue plasminogen activator (tPA)-related cleavage of CDCP1 [8]. Therapeutic inhibition of ADAM9, including RNA interference-based silencing, markedly reduces tumor development, invasion and motility in non-small cell lung cancer (NSCLC) models, underscoring its promise as a therapeutic target [9]. These results highlight the essential function of ADAM9 in the progression of lung cancer and its promise as a target for treatment.

Non-coding RNAs, including circular RNAs (circRNAs) and microRNAs (miRNAs), are critical mediators of gene synthesis at both transcriptional and post-transcriptional levels. They function as oncogenic or tumor-suppressive agents across diverse cancer types [10,11]. CircRNAs often act as durable molecular sponges for miRNAs, thereby modulating their functions and indirectly regulating oncogenes and tumor suppressors [10,12,13]. The circRNA–miRNA regulatory networks are now recognized as crucial components of oncogenic signaling pathways and represent promising avenues for targeted therapeutic development.

Bone sialoprotein (BSP), part of the small integrin-binding ligand N-linked glycoprotein (SIBLING) family, has long been considered correlated with the aggressiveness of tumors. BSP promotes malignant cell growth, detachment, migration, and metastatic colonization [14]. Its expression is particularly enriched in bone metastases relative to visceral metastases, consistent with a role in organ-specific tropism [15]. Elevated BSP levels have also been linked to metastasis and poorer outcomes in NSCLC [16,17]. These results highlight BSP as an essential mediator of tumor progression and a potential medical target in metastatic conditions. This research illustrates that BSP facilitates the proliferation and spread of lung cancer by inducing ADAM9 expression through the hsa_circ_0084050/miR-373-3p axis. Our findings reveal a previously unrecognized mechanism to target BSP that potentially inhibits lung cancer progression and metastasis.

## 2. Results

### 2.1. Elevated BSP Expression Is Associated with Lung Cancer Progression and Metastasis

**Figure 1:**
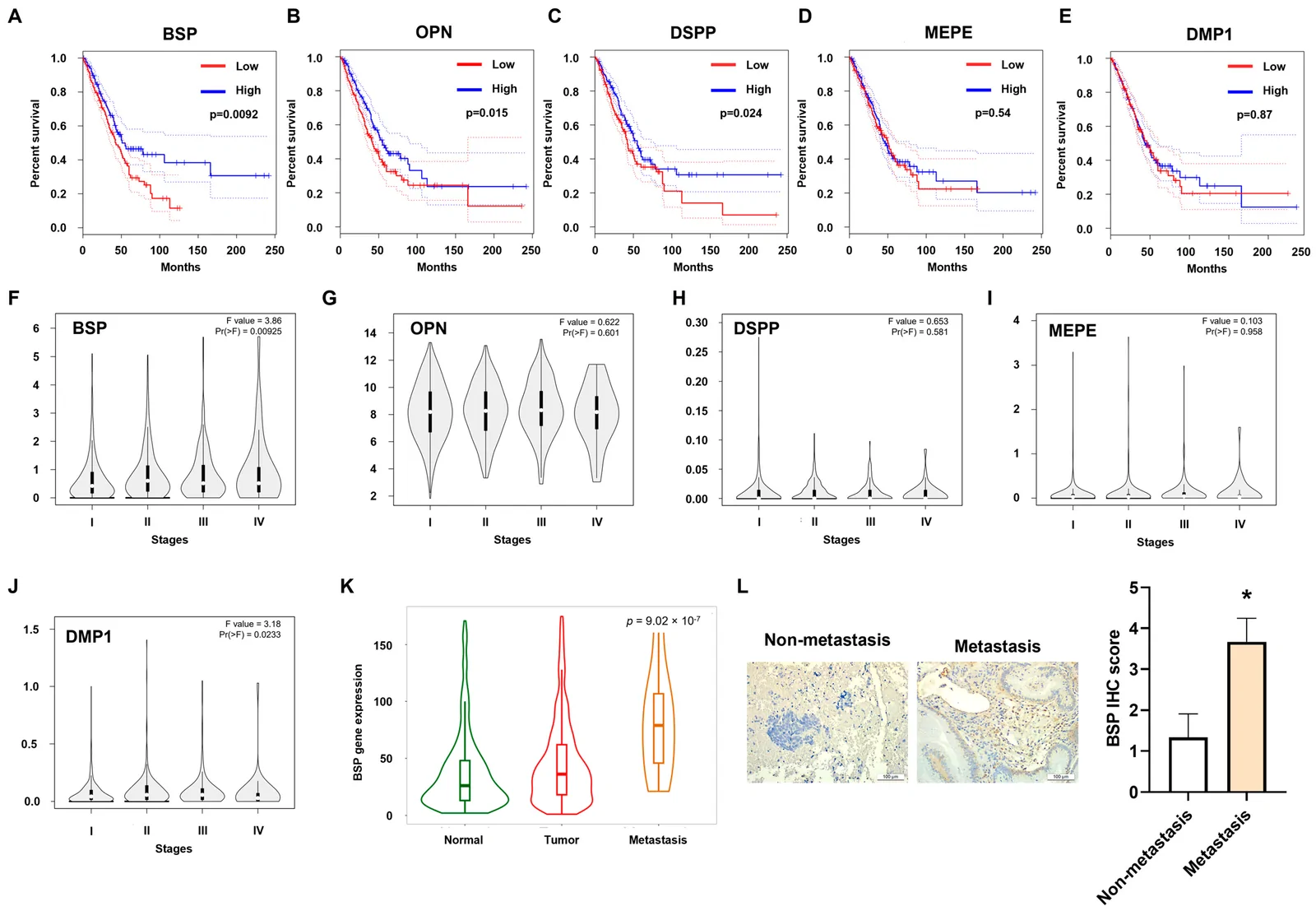
Expression of BSP in lung cancer patients. (**A**–**E**) The overall survival rates of lung cancer patients based on the elevated or lower expression of certain SIBLING family genes. Red and blue solid lines represent the low- and high-expression groups, respectively. (**F**–**J**) Stage-dependent expression patterns of SIBLING family members in lung cancer, analyzed using GEPIA2. (**K**) Analysis of BSP expression throughout normal lung tissue, initial lung tumors, and metastatic lesions by TNMplot. (**L**) BSP expression in lung cancer specimens from non-metastatic and metastatic patients (*n* = 3 independent patients per group). Data are presented as mean ± SD and were analyzed using an unpaired two-tailed Student’s *t*-test. * *p* < 0.05 compared with the non-metastatic group.

**Figure 2:**
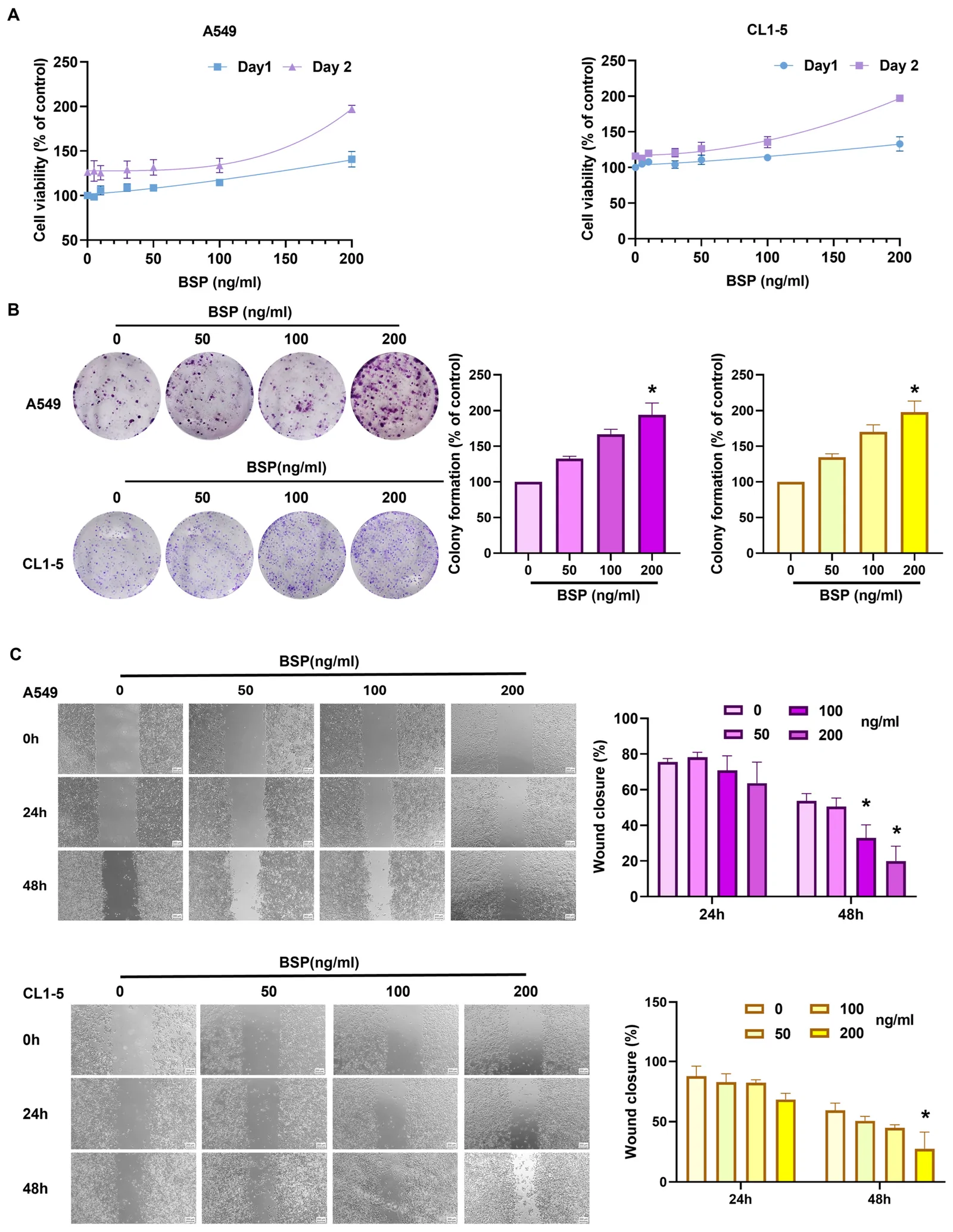
BSP promotes the proliferation and motility of lung cancer cells. (**A**) BSP-induced changes in cell viability at 24 and 48 h at the indicated concentrations, assessed by the MTT assay. (**B**) Dose-dependent effects of BSP on colony formation in A549 and CL1-5 cells. (**C**) Cell migration following treatment with the indicated concentrations of BSP, evaluated by wound-healing assay at 24 and 48 h. Data are presented as mean ± SD from three independent biological experiments (*n* = 3). Statistical significance was determined by one-way ANOVA followed by Dunnett’s multiple-comparisons test. For panels (**A**–**C**), each BSP-treated group was compared with the corresponding untreated control (0 ng/mL) within the same cell line and time point. * *p* < 0.05 versus the corresponding control.

SIBLING family proteins are overexpressed in various tumors and are regulated in critical steps such as cancer cell adhesion, proliferation, migration, metastasis, and angiogenesis [18]. Investigation of the GEPIA2 database indicated no significant differences in overall survival (OS) among individuals exhibiting high or low expression levels of certain SIBLING family members, such as dentin matrix acidic phosphoprotein 1 (DMP1) and matrix extracellular phosphoglycoprotein (MEPE). Conversely, those with heightened levels of BSP, osteopontin (OPN), or dentin sialophosphoprotein (DSPP) demonstrated significantly reduced OS in comparison to those with lower expression (Figure 1A–E). GEPIA database analysis further demonstrated stage-dependent alterations in BSP expression (Figure 1F–J). Consistently, TNMplot data indicated that BSP levels were elevated in metastatic tissues relative to original tumors and healthy controls (Figure 1K). These findings were corroborated by clinical samples, in which BSP expression is higher in metastatic lung cancer compared with non-metastatic cases (Figure 1L). The collective expression of BSP is critical for the progression and widespread dissemination of lung cancer. Administering escalating concentrations of BSP to lung cancer cells (A549 and CL1-5) led to a concentration-dependent increase in cell viability (Figure 2A). BSP stimulation also significantly enhanced both proliferation and motility in these cell lines (Figure 2B,C). Thus, BSP promotes proliferation and motility in lung cancer cells.

### 2.2. BSP Stimulates ADAM9-Dependent Lung Cancer Cell Proliferation and Mobility

**Figure 3:**
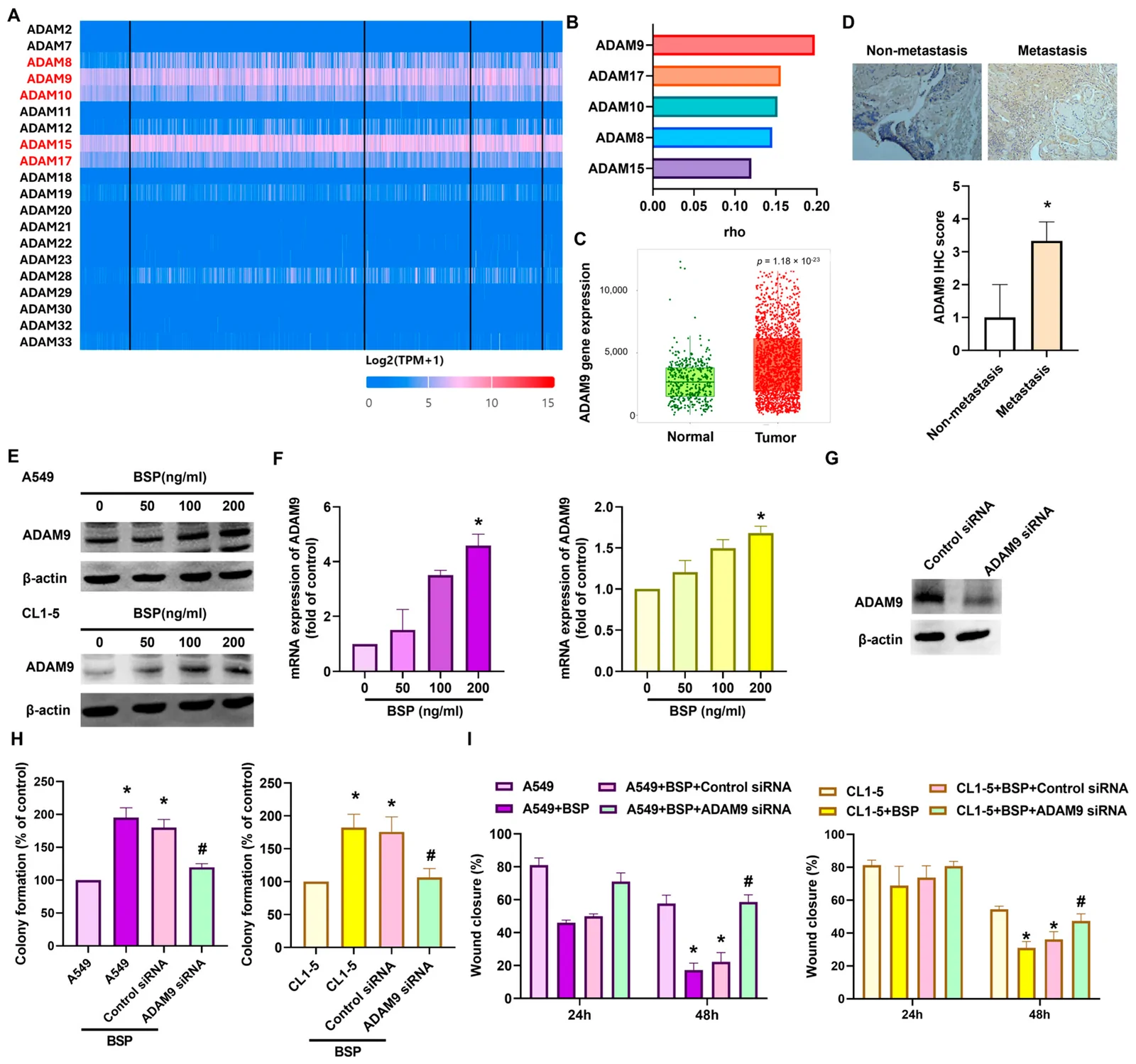
BSP stimulates the expression of ADAM9 in lung carcinoma cells. (**A**) ADAM family gene expression profiles in lung adenocarcinoma were examined using the TCGA-LUAD dataset. ADAM8, ADAM9, ADAM10, ADAM15, and ADAM17, shown in red, were selected for subsequent correlation analysis. (**B**) Correlations between BSP and individual ADAM genes were evaluated through TIMER2.0. (**C**) ADAM9 expression in lung cancer patients from the TNMplot database. (**D**) ADAM9 expression in lung cancer specimens from non-metastatic and metastatic patients (*n* = 3 independent patients per group); statistical significance was determined using an unpaired two-tailed Student’s *t*-test. (**E**,**F**) ADAM9 protein and mRNA expression following treatment with the indicated concentrations of BSP. For panel (**F**), data are presented as mean ± SD from three independent biological experiments (*n* = 3) and were analyzed using one-way ANOVA followed by Dunnett’s multiple-comparisons test, with each BSP-treated group compared with the corresponding untreated control. (**G**) Verification of ADAM9 knockdown efficiency in A549 cells following transfection with ADAM9 siRNA compared to control siRNA. (**H**,**I**) Effects of ADAM9 silencing on BSP-induced colony formation and cell migration. Data are presented as mean ± SD from three independent biological experiments (*n* = 3) and were analyzed using one-way ANOVA followed by Tukey’s multiple-comparisons test. Planned comparisons included untreated control versus BSP-treated cells and BSP + control siRNA versus BSP + ADAM9 siRNA. * *p* < 0.05 versus the corresponding control; # *p* < 0.05 versus the BSP + control siRNA group.

ADAM family proteins have been widely implicated in promoting tumor metastasis [19,20,21]. Transcriptomic analysis of TCGA lung cancer datasets revealed that ADAM8, ADAM9, ADAM10, ADAM15, and ADAM17 were expressed at high levels (Figure 3A). Correlation analysis using TIMER 2.0 demonstrated the strongest positive association between BSP and ADAM9 expression (Figure 3B). The expression of ADAM9 was markedly increased in tumor tissues compared with normal controls (Figure 3C) and validated by IHC staining in clinical samples (Figure 3D). Western blotting and qPCR confirmed that BSP treatment increased ADAM9 expression in both A549 and CL1-5 cells (Figure 3E,F). Functional assays further showed that silencing ADAM9 expression attenuated BSP-induced proliferation and motility (Figure 3G–I). Together, these results reveal that BSP enhances lung cancer cell proliferation and motility through upregulation of ADAM9.

### 2.3. BSP Enhances ADAM9-Dependent Proliferation and Motility via Activation of the FAK Pathway

**Figure 4:**
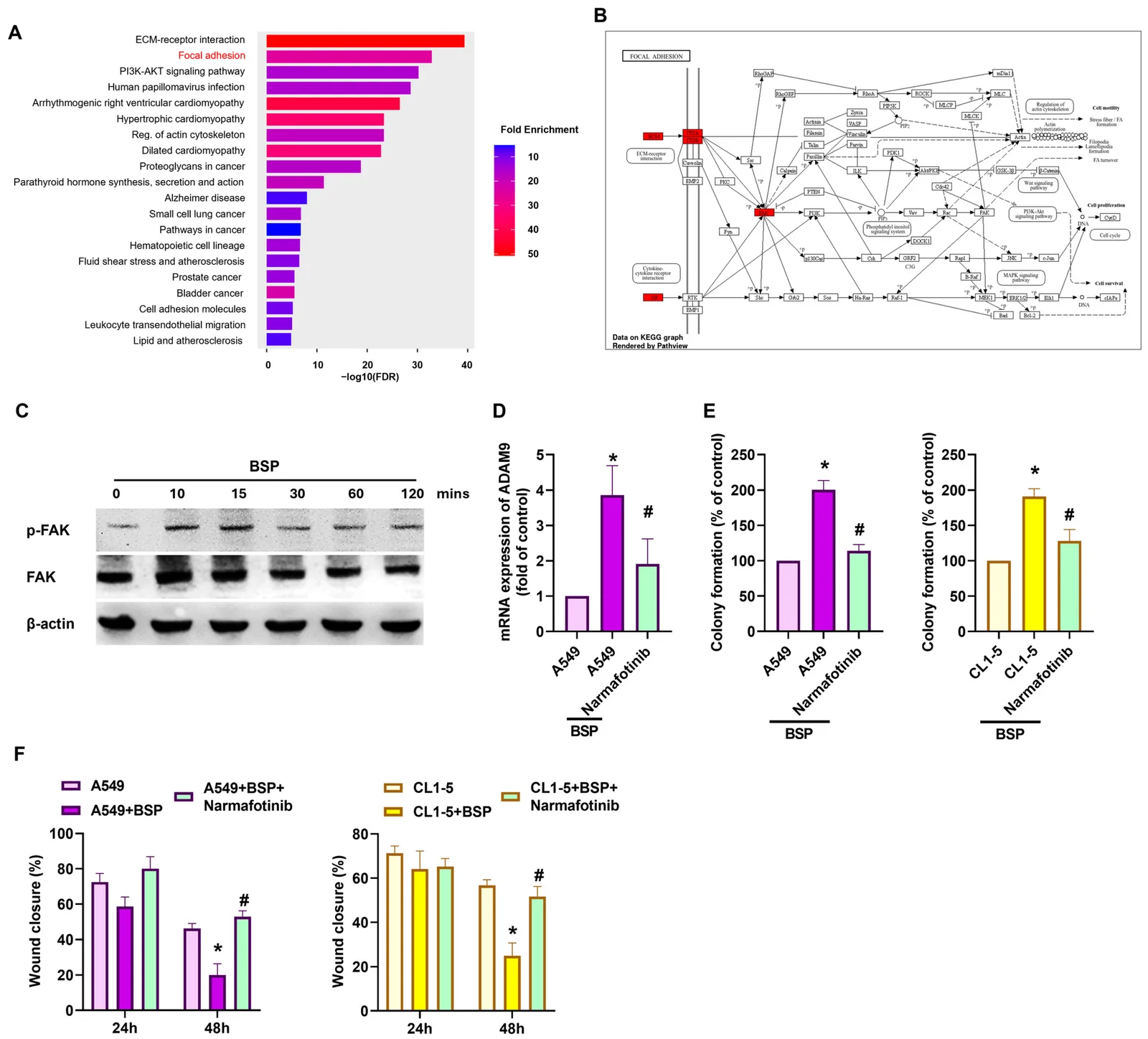
FAK signaling plays a role in the induction of ADAM9 expression by BSP and promotes the proliferation and mobility of lung cancer cells. (**A**,**B**) Pathway enrichment analysis identifying signaling pathways significantly elevated in BSP-treated cells. In panel (**A**), focal adhesion, highlighted in red, was selected for further analysis. In panel (**B**), solid and dashed arrows indicate direct and indirect regulatory interactions, respectively. (**C**) Time-dependent phosphorylation of FAK in BSP-stimulated cells, determined by Western blotting. (**D**) ADAM9 mRNA levels in A549 cells exposed to BSP with or without the FAK inhibitor narmafotinib, quantified using RT-qPCR. (**E**,**F**) Treatment with narmafotinib in A549 and CL1-5 cells markedly reduced BSP-stimulated colony formation and cell motility, as shown by colony formation and wound-healing experiments, respectively. Cells were pretreated with narmafotinib (0.1 μM) for 30 min before BSP stimulation. Data are presented as mean ± SD from three independent biological experiments (*n* = 3). For panels (**D**–**F**), statistical significance was determined by one-way ANOVA followed by Tukey’s multiple-comparisons test. Planned comparisons included untreated control versus BSP-treated cells and BSP versus BSP+ Narmafotinib. * *p* < 0.05 versus the corresponding control; # *p* < 0.05 versus the BSP-treated group.

ADAM9 was reported to regulate the development and aggressiveness of lung cancer, partly by modulating metastatic pathways [8,22,23]. To investigate the pathway involved in BSP influences ADAM9 expression and cell function, pathway analysis was performed, revealing a significant correlation with focal adhesion mechanisms and FAK activity (Figure 4A,B). Western blotting demonstrated that BSP rapidly induced FAK phosphorylation in A549 cells within 10 min of treatment (Figure 4C). Importantly, treatment with the FAK inhibitor (narmafotinib) reduced BSP-induced ADAM9 expression (Figure 4D), as well as BSP-mediated increases in proliferation and motility in both cell lines (Figure 4E,F). To further evaluate the involvement of FAK using a genetic approach, FAK was silenced using siRNA in A549 and CL1-5 cells. FAK silencing significantly attenuated BSP-induced ADAM9 mRNA expression and wound healing in both cell lines (Supplementary Figure S1), further supporting the involvement of FAK in BSP-mediated ADAM9 regulation and cellular motility (Supplementary Figure S1). These findings indicate that BSP enhances ADAM9 synthesis and enhances lung cancer cell proliferation and motility through FAK activation.

### 2.4. miR-373-3p Regulates BSP-Induced ADAM9 Expression as Well as Cell Proliferation and Mobility

**Figure 5:**
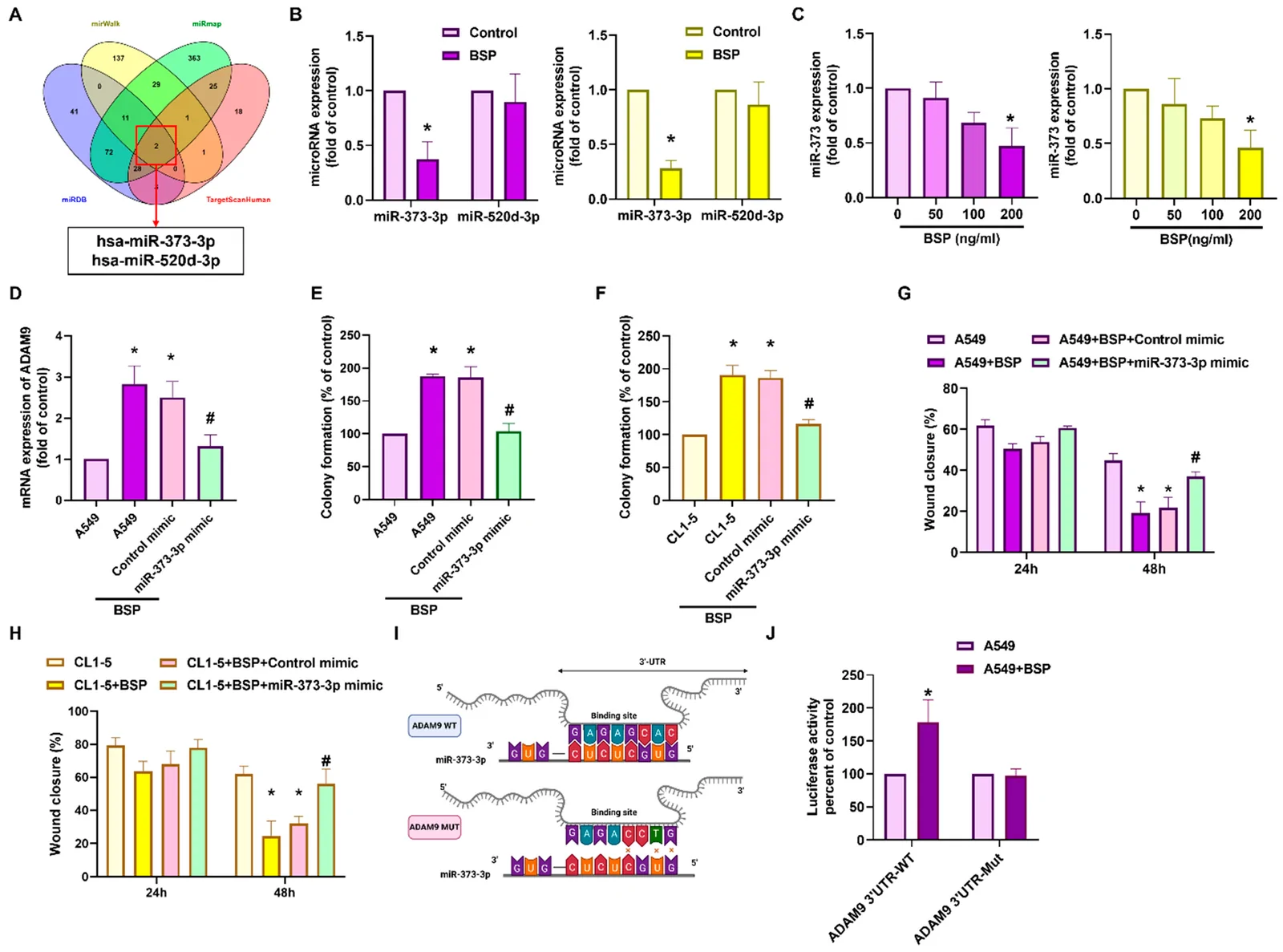
BSP promotes the proliferation and motility of lung cancer cells by suppressing miR-373-3p expression. (**A**) Predicted miRNAs targeting ADAM9 identified through combined analysis of four databases. (**B**) Expression of the indicated miRNAs following BSP treatment, determined by RT-qPCR. Each miRNA was analyzed separately using an unpaired two-tailed Student’s *t*-test. (**C**) Dose-dependent effects of BSP on miR-373-3p expression. Statistical significance was determined by one-way ANOVA followed by Dunnett’s multiple-comparisons test, with each BSP-treated group compared with the untreated control. (**D**) ADAM9 mRNA expression following transfection with miR-373-3p mimic. (**E**,**F**) Effects of miR-373-3p overexpression on BSP-induced colony formation in A549 and CL1-5 cells. (**G**,**H**) Effects of miR-373-3p overexpression on BSP-induced cell migration. For panels (**D**–**H**), statistical significance was determined by one-way ANOVA followed by Tukey’s multiple-comparisons test. Planned comparisons included untreated control versus BSP and BSP + control mimic versus BSP + miR-373-3p mimic. (**I**) Schematic representation of the predicted miR-373-3p binding site within the ADAM9 3′-UTR. (**J**) Relative luciferase activity of WT and MUT ADAM9 3′-UTR reporters following BSP treatment; statistical significance was determined by one-way ANOVA followed by Tukey’s multiple-comparisons test. Data are presented as mean ± SD from three independent biological experiments (*n* = 3). * *p* < 0.05 versus the corresponding control; # *p* < 0.05 versus the BSP + control mimic group.

miRNAs play crucial roles in regulating lung cancer metastasis and development [24,25]. Bioinformatic screening using four databases (miRWalk, TargetScan, miRDB, miRmap) indicated miR-373-3p as a potential regulator of ADAM9 (Figure 5A). BSP treatment significantly reduced miR-373-3p expression compared with controls (Figure 5B), with a concentration-dependent decline observed in both cell lines (Figure 5C). Transfection with a miR-373-3p mimic effectively suppressed BSP-promoted ADAM9 expression in A549 cells (Figure 5D), and concomitantly inhibited BSP-mediated proliferation and motility (Figure 5E–H). Luciferase reporter assays further indicated that miR-373-3p directly interacts with the ADAM9 3′-UTR, as BSP enhanced activity of the wild-type but not mutant ADAM9 3′-UTR reporter (Figure 5I,J). These findings indicate that BSP enhances lung cancer cell proliferation and movement by upregulating ADAM9 through suppression of miR-373-3p.

### 2.5. BSP Regulates ADAM9 Expression and Cell Proliferation and Migration Through hsa_circ_0084050 Sponging of miR-373-3p

**Figure 6:**
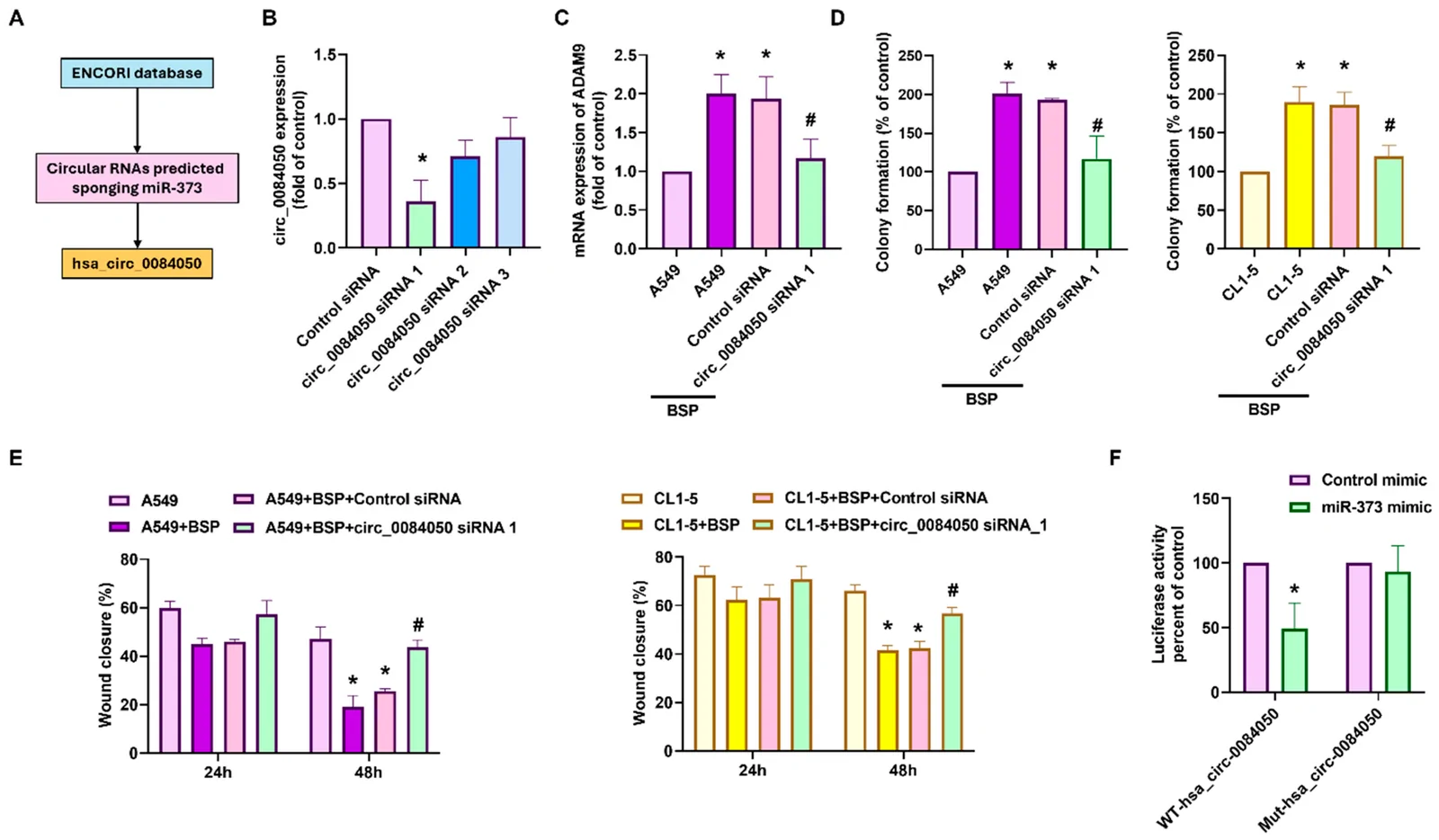
BSP enhances lung cancer cell proliferation and mobility by increasing ADAM9 expression through upregulation of hsa_circ_0084050, which sponges miR-373-3p. (**A**) Prediction of circRNAs potentially interacting with miR-373-3p using the ENCORI database. (**B**) Silencing efficiency of three independent siRNAs targeting hsa_circ_0084050 in A549 cells. Statistical significance was determined by one-way ANOVA followed by Dunnett’s multiple-comparisons test, with each hsa_circ_0084050 siRNA group compared with the control siRNA group. (**C**) ADAM9 mRNA expression following hsa_circ_0084050 silencing and BSP treatment. (**D**) Effects of hsa_circ_0084050 silencing on BSP-induced colony formation in A549 and CL1-5 cells. (**E**) Effects of hsa_circ_0084050 silencing on BSP-induced cell migration. For panels (**C**–**E**), statistical significance was determined by one-way ANOVA followed by Tukey’s multiple-comparisons test. Planned comparisons included untreated control versus BSP and BSP + control siRNA versus BSP + hsa_circ_0084050 siRNA 1. (**F**) Relative luciferase activity of WT- or MUT-hsa_circ_0084050 reporter constructs following co-transfection with control mimic or miR-373-3p mimic; statistical significance was determined by one-way ANOVA followed by Tukey’s multiple-comparisons test. Data are presented as mean ± SD from three independent biological experiments (*n* = 3). * *p* < 0.05 versus the corresponding control; # *p* < 0.05 versus the BSP + control siRNA group.

CircRNAs are progressively acknowledged as modulators of cancer progression by acting as miRNA sponges [26,27,28]. Bioinformatic analysis using the starBase database predicted hsa_circ_0084050 as a potential circRNA capable of binding miR-373-3p (Figure 6A). hsa_circ_0084050 has previously been annotated in circBase as a circular transcript derived from the ADAM9 locus, with reported circular-junction evidence [29,30]. To investigate its role in lung cancer, three siRNAs targeting hsa_circ_0084050 were designed. The transfection of these siRNAs into cells significantly decreased the hsa_circ_0084050 expression, with siRNA 1 demonstrating the highest inhibitory efficacy (Figure 6B). Moreover, hsa_circ_0084050 siRNA 1 reversed the BSP-mediated upregulation of ADAM9 mRNA expression (Figure 6C). Consistent effects were observed in both A549 and CL1-5 cells; transfection with hsa_circ_0084050 siRNA 1 significantly attenuated BSP-induced cell proliferation and migration (Figure 6D,E). To further examine the predicted interaction between hsa_circ_0084050 and miR-373-3p, luciferase reporter constructs containing either the wild-type (WT-hsa_circ_0084050) or mutant (MUT-hsa_circ_0084050) miR-373-3p-binding sequence were generated. Co-transfection with the miR-373-3p mimic significantly reduced luciferase activity of the WT reporter, whereas no significant change was observed with the mutant reporter (Figure 6F). These findings support a sequence-dependent interaction between hsa_circ_0084050 and miR-373-3p. Together, these data suggest that BSP promotes ADAM9 expression and the associated proliferation and cell mobility, at least in part, through regulation of the hsa_circ_0084050/miR-373-3p axis.

### 2.6. BSP Knockdown Inhibits Lung Cancer Progression and Dissemination in a Murine Model

**Figure 7:**
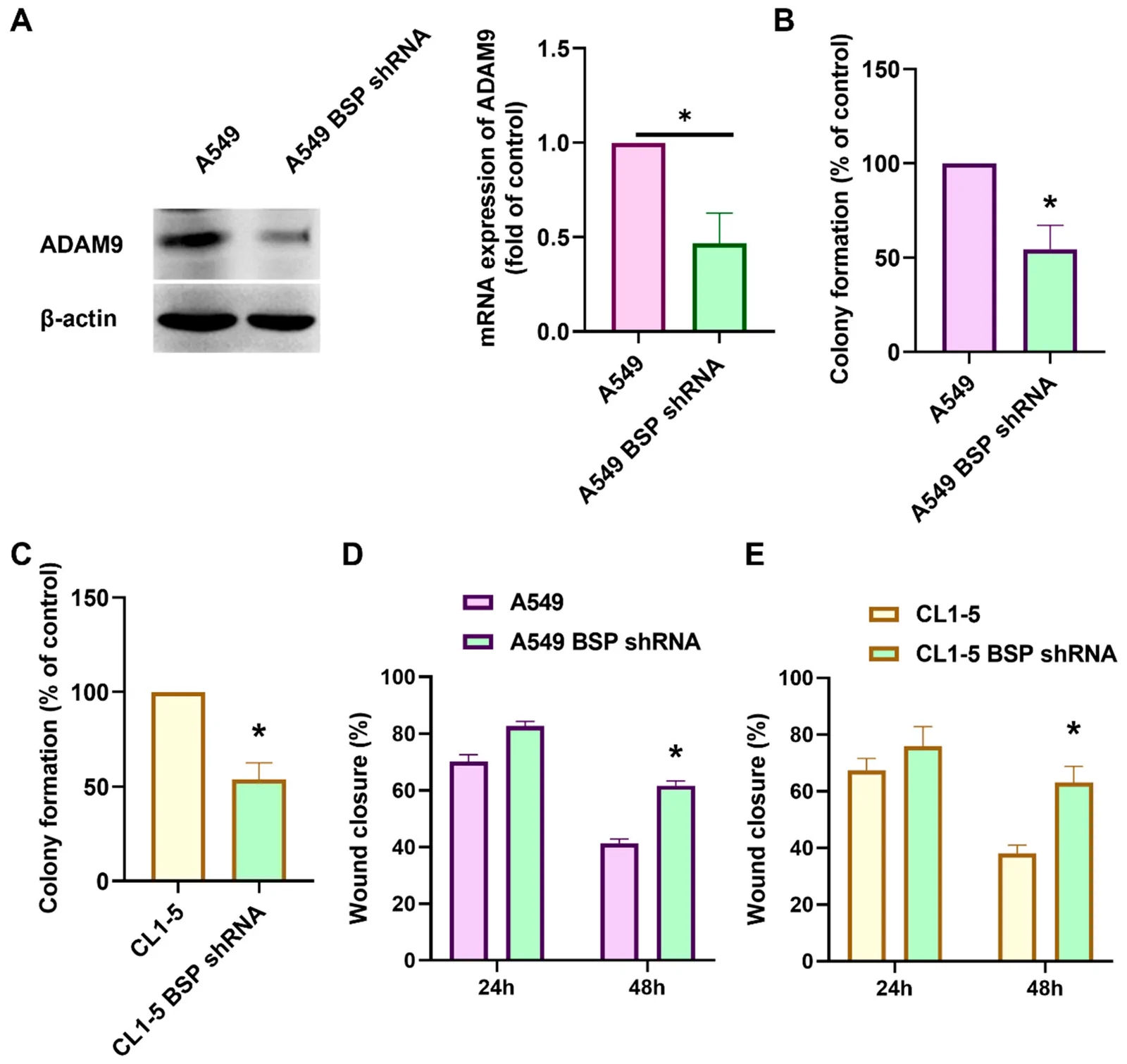
BSP knockdown reduces ADAM9 expression and suppresses lung cancer cell proliferation and mobility. (**A**) ADAM9 protein and mRNA expression in control A549 cells and the previously validated stable A549 BSP-shRNA clone. (**B**,**C**) Effects of BSP knockdown on colony formation in A549 and CL1-5 cells. (**D**,**E**) Effects of BSP knockdown on cell migration in A549 and CL1-5 cells, evaluated by wound-healing assay at 24 and 48 h. Data are presented as mean ± SD from three independent biological experiments (*n* = 3). Statistical significance was determined using an unpaired two-tailed Student’s *t*-test; for panels (**D**,**E**), and comparisons were performed separately at each time point. * *p* < 0.05 versus the corresponding control.

**Figure 8:**
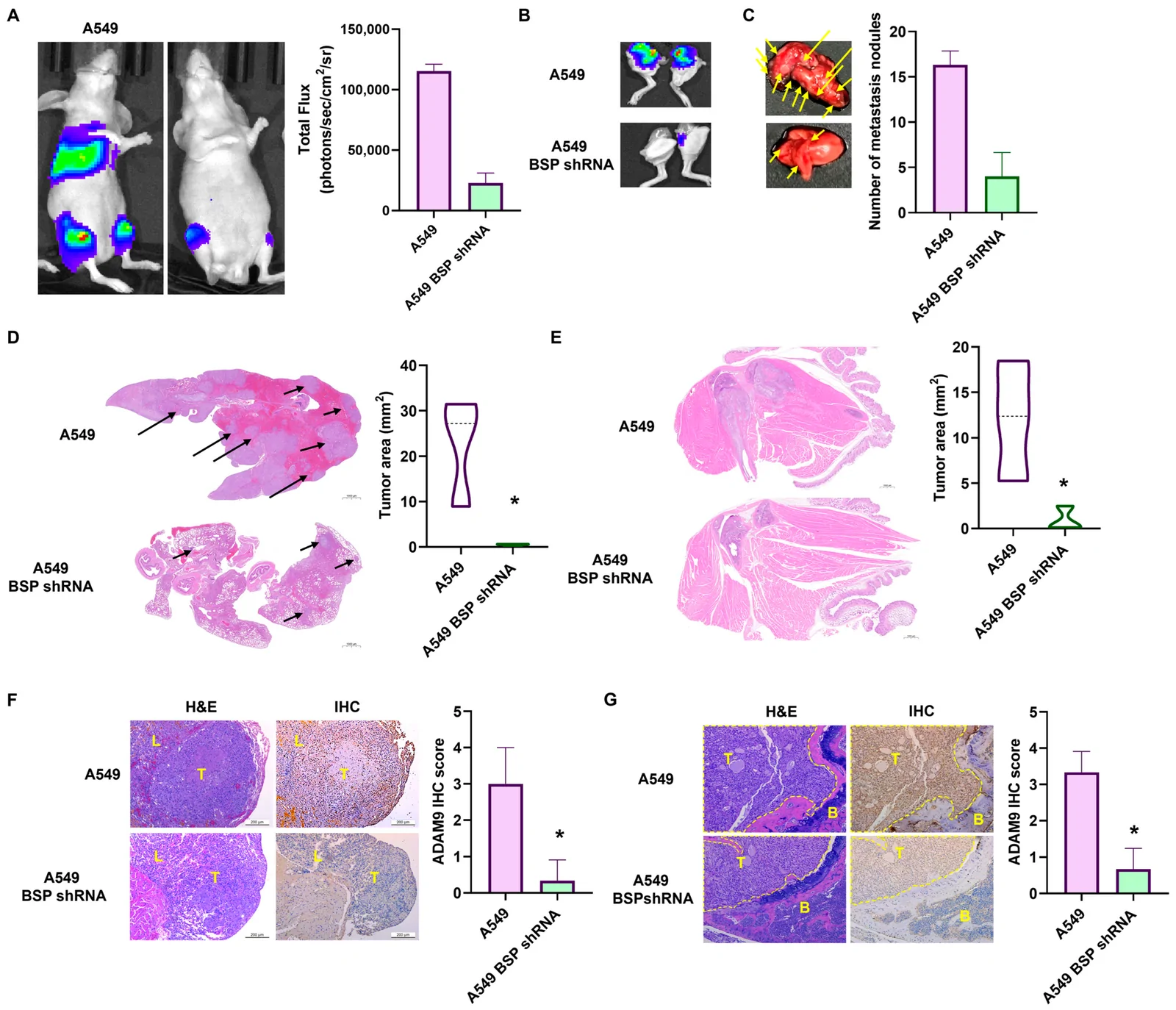
BSP knockdown is associated with reduced lung cancer progression and metastasis in an exploratory animal model. (**A**) A549 cells or stable A549 BSP-shRNA cells were injected into the caudal artery of BALB/c nude mice, and metastatic burden was evaluated by bioluminescence imaging. The colored areas indicate bioluminescence signal intensity. (**B**,**C**) Ex vivo imaging of hindlimbs and lungs and quantification of metastatic nodules. In panel (**C**), yellow arrows indicate metastatic nodules in the lung. (**D**,**E**) Histological assessment and quantification of metastatic tumor area in lung and hindlimb tissues. The dashed lines indicate the median values. (**F**,**G**) H&E and IHC analyses of ADAM9 expression in lung and hindlimb lesions. In panel (**G**), the yellow dashed lines in the H&E images and in the IHC images delineate the tumor–bone interface. B, bone; L, lung tissue; T, tumor. Data are presented as mean ± SD from three mice per group (*n* = 3). Statistical significance was determined using an unpaired two-tailed Student’s *t*-test comparing A549 with A549 BSP-shRNA mice. * *p* < 0.05 versus the A549 control group.

To validate these findings in vivo, a previously established stable A549 BSP-shRNA cell line was used. BSP knockdown at the protein level in this cell line was previously validated in our published study [31]. Western blotting and qPCR demonstrated markedly reduced ADAM9 expression in BSP-knockdown cells compared with controls (Figure 7A). BSP silencing also decreased colony formation (Figure 7B,C) and motility in vitro (Figure 7D,E). In a mouse metastasis model, injection of A549 BSP-shRNA cells via the caudal artery resulted in significantly lower bioluminescence signals in metastatic sites after 8 weeks compared with mice injected with control A549 cells (Figure 8A). Ex vivo imaging of legs (Figure 8B) and lungs (Figure 8C) confirmed reduced metastasis in the BSP-knockdown group. Histological analysis revealed smaller tumor areas in the BSP-knockdown group compared with controls (Figure 8D,E), and IHC staining confirmed reduced ADAM9 expression in BSP-knockdown tumors (Figure 8F,G). Together, these findings suggest that BSP knockdown markedly inhibits lung cancer growth and metastasis in vivo.

## 3. Discussion

Lung cancer is a primary factor in global cancer mortality, with its high lethality attributed mainly to the aggressive nature of the disorder and its strong propensity for metastasis [32]. Dissemination to distant sites is the primary driver of cancer-associated deaths, and once distant spread occurs, lung cancer is frequently incurable with current systemic therapies [5,33]. The biological mechanisms that enable metastasis are complex, involving multiple signaling networks and regulatory molecules [4,34]. Previous reports have shown that BSP promotes lung cancer metastasis by inducing MMP14 expression [31,35]. In the present investigation, we found that BSP upregulates ADAM9 expression, thereby enhancing lung cancer cell proliferation and motility. Mechanistically, BSP regulates ADAM9 expression through activation of the FAK signaling pathway and modulation of the hsa_circ_0084050/miR-373-3p axis. These findings collectively suggest that BSP may represent a novel therapeutic target for lung cancer progression and metastasis.

BSP, a member of the SIBLING family of matricellular proteins, is increasingly recognized as a key regulator of tumor progression and metastatic dissemination [14,36,37]. Elevated circulating BSP levels have also been proposed as a biomarker for early detection of bone metastases and as a prognostic factor for metastatic burden [38]. In breast cancer, BSP expression is closely associated with bone metastasis, and its detection in primary tumors predicts skeletal involvement [36,39]. We confirmed that BSP has highly increased expression in lung cancer. Similarly, in lung cancer, BSP has been reported to promote anoikis resistance through reduction in miR-150-5p and activation of the ERK pathway [35]. Our results show that BSP enhances lung cancer cell colony formation and migration. Importantly, BSP knockdown reduced tumor burden and dissemination in vivo, further supporting the function of BSP in lung cancer progression and metastasis. However, the small sample size, use of only male mice and a single A549 xenograft model in immunodeficient animals, and potential variability in bioluminescence imaging may limit the precision and generalizability of these findings.

Metastasis is a multistep process involving detachment of tumor cells, invasion into surrounding tissues, dissemination through the circulation, and colonization of distant organs [40,41]. This process is frequently facilitated by epithelial–mesenchymal transition (EMT), which enhances migratory and invasive properties [41,42]. ADAM9, a member of the disintegrin and metalloproteinase family, has been implicated in cancer progression and poor prognosis across multiple tumor types [7,43]. In lung adenocarcinoma, high ADAM9 expression correlates with poor outcomes and increased angiogenesis, whereas ADAM9 downregulation reduces tumor growth and vascularization [44]. Here, we found that ADAM9 expression is positively associated with BSP levels in clinically analyzed lung cancer samples and is markedly elevated in metastatic tissues. However, the clinical specimen analysis was based on a limited cohort of six patients and a single representative tumor field per specimen, which may increase susceptibility to sampling bias and limit the generalizability of these findings. Therefore, the clinical observations should be considered exploratory and require validation in larger independent cohorts using multi-field quantitative assessment. Silencing ADAM9 expression significantly inhibited BSP-induced proliferation and migration in vitro, while BSP knockdown suppressed ADAM9 expression in vivo. These results establish that BSP promotes tumor progression and metastasis through an ADAM9-dependent mechanism. Although BSP knockdown reduced both ADAM9 expression and metastatic burden in vivo, the present study did not include restoration of ADAM9 in BSP-deficient cells. Therefore, these findings do not establish that the in vivo phenotype is mediated specifically through ADAM9, and future rescue studies will be required to clarify this causal relationship.

In recent times, circRNAs have been identified as significant modulators of cancer initiation and progression [45,46,47]. Acting as stable RNA molecules, circRNAs often serve as sponges for miRNAs, thereby relieving suppression of oncogenic targets [48,49,50]. For example, circRNAs have been found to control EMT, proliferation, and metastasis in several cancers, including head and neck cancer, where they are being actively explored as therapeutic targets [51]. In the present investigation, we identified hsa_circ_0084050 as an essential mediator of BSP-induced ADAM9 expression. Silencing hsa_circ_0084050 with siRNA reversed the BSP-induced proliferation and migration of lung cancer cells. Moreover, BSP treatment led to a reduction in miR-373-3p levels, while functional assays confirmed that hsa_circ_0084050 directly binds miR-373-3p, relieving its inhibitory effect on ADAM9. This interaction forms a regulatory loop—the BSP–hsa_circ_0084050/miR-373-3p–ADAM9 axis—that promotes lung cancer cell proliferation, motility, and metastatic potential. Although the wild-type/mutant luciferase reporter assay and functional silencing experiments support a regulatory relationship between hsa_circ_0084050 and miR-373-3p, these approaches do not directly demonstrate their endogenous physical interaction. Therefore, whether hsa_circ_0084050 functions as an endogenous sponge for miR-373-3p should be further examined using RNA pull-down or AGO2-RIP assays. hsa_circ_0084050 has previously been identified and annotated in circBase as a circular transcript derived from the ADAM9 locus, with supporting circular-junction sequencing evidence reported in the Salzman et al. dataset [29,30]. Nevertheless, its circular identity was not independently validated in the A549 and CL1-5 cells used in the present study by divergent PCR, back-splice junction sequencing, or RNase R digestion. Therefore, additional structural validation in lung cancer cells will be required to further confirm its circular identity.

FAK is a non-receptor tyrosine kinase that controls multiple cellular processes, such as organ size control, tissue regeneration, tumorigenesis, and metastasis [52,53,54]. Its activation enhances migration, invasion, angiogenesis, and EMT, and involves the maintenance of cancer stem cell properties [53,54]. Moreover, FAK signaling is known to interact with pathways such as Wnt, further shaping tumorigenic processes in a context-dependent manner [55]. Overexpression of FAK has been indicated in several malignancies, such as prostate, colorectal, and liver cancers, and is strongly correlated with aggressive phenotypes and poor survival [52,54,56]. In this study, pathway enrichment analyses using the STRING and ShinyGO databases revealed a significant association between FAK signaling and ADAM9 expression in the context of BSP induction. Consistent with these bioinformatic findings, our experimental data demonstrated that BSP treatment led to FAK phosphorylation, while pharmacological inhibition of FAK markedly suppressed BSP-induced ADAM9 upregulation, as well as the enhanced cell proliferation and motility. Importantly, genetic silencing of FAK also attenuated BSP-induced ADAM9 expression and cell migration in both A549 and CL1-5 cells. Together, the pharmacological and genetic evidence supports an important functional role of FAK in BSP-mediated ADAM9 regulation in lung cancer cells. Taken together, our findings suggest that FAK signaling is a crucial mediator of BSP-induced ADAM9 expression and function in lung cancer cells.

**Figure 9:**
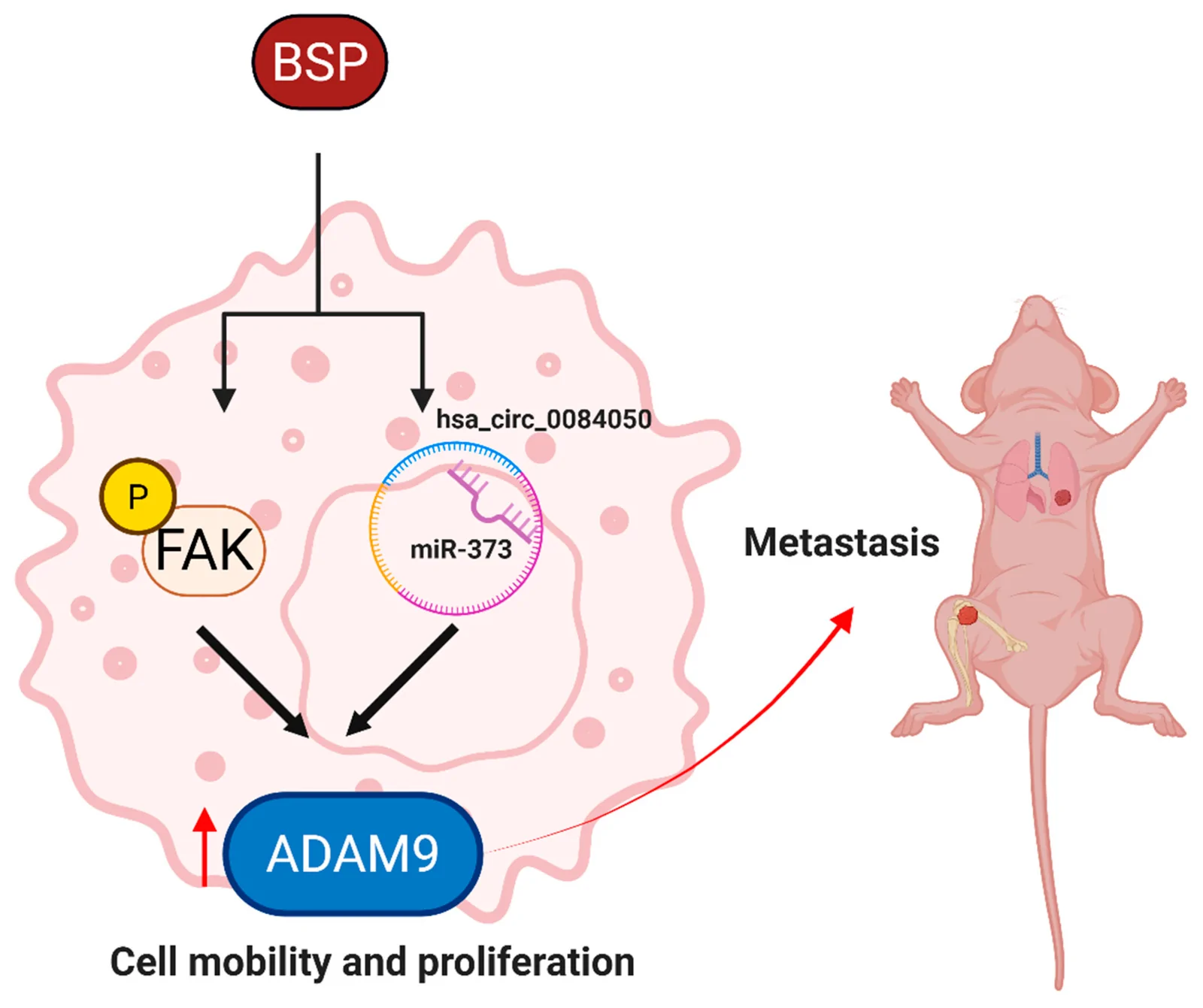
Schematic illustration of BSP involvement in lung cancer progression and metastasis. BSP promotes ADAM9-dependent proliferation and migration in lung cancer cells by activating FAK signaling and modulating the hsa_circ_0084050/miR-373-3p regulatory axis. Different colors are used to distinguish the molecular components of the schematic. Black arrows indicate regulatory relationships, whereas red arrows indicate increased ADAM9 expression and its downstream promotion of metastasis. The multicolored circular structure represents hsa_circ_0084050, and the purple structure represents miR-373-3p; the different colors are used for illustrative purposes only.

In summary, our findings support a role for BSP in lung cancer progression and metastasis through activation of FAK signaling and modulation of the hsa_circ_0084050/miR-373-3p axis to regulate ADAM9 expression (Figure 9). Although these findings suggest that BSP may represent a candidate molecular target for further investigation, additional pharmacological, safety, efficacy, and clinical studies are required before its therapeutic potential can be established.

## 4. Materials and Methods

### 4.1. Materials

Recombinant BSP protein was sourced from R&D Systems located in Minneapolis, MN, USA. Small interfering RNAs (siRNAs) directed targeting ADAM9 and control siRNAs were acquired from Santa Cruz Biotechnology (Santa Cruz, CA, USA). BSP shRNA constructs were supplied by the National RNAi Core Facility (Academia Sinica, Taipei, Taiwan). Three independent siRNAs specific to hsa_circ_0084050 were purchased from MDBio Inc. (Taipei, Taiwan). Lipofectamine™ 2000 and the miR-373 mimic were obtained from Thermo Fisher Scientific (Waltham, MA, USA). Reporter lysis buffer was obtained from Promega (Madison, WI, USA). Primary antibodies included anti-ADAM9 (GTX130081, GeneTex, Hsinchu, Taiwan), anti-BSP (PA1505, Boster Biological Technology, Pleasanton, CA, USA), anti-p-FAK (Y397, Cell Signaling Technology, Danvers, MA, USA), anti-FAK (SC-271195, Santa Cruz Biotechnology), and anti-β-actin (A5441, Sigma-Aldrich, St. Louis, MO, USA). HRP-conjugated secondary antibodies were sourced from Santa Cruz Biotechnology. Narmafotinib (AMP-945; MedChemExpress, Monmouth Junction, NJ, USA) was used at 0.1 μM with a 30 min pretreatment before BSP stimulation, based on its reported potency and kinase selectivity for FAK [57].

### 4.2. Cell Lines and Culture Conditions

A549 human non-small cell lung cancer cells were obtained from the American Type Culture Collection (ATCC CCL-185; Manassas, VA, USA; Cellosaurus accession no. CVCL_0023) and maintained in RPMI-1640 medium containing 10% fetal bovine serum (FBS). CL1-5 human lung adenocarcinoma cells (Cellosaurus accession no. CVCL_D521) were generously supplied by Dr. Shun-Fa Yang of Chung Shan Medical University (Taichung, Taiwan) and propagated in Dulbecco’s modified Eagle’s medium supplemented with 10% FBS. Both cell lines were cultured at 37 °C in a humidified atmosphere containing 5% CO_2_ and were passaged or collected for experiments at approximately 80% confluence. To establish stable BSP-knockdown cells, A549 cells were transduced with lentiviral particles encoding a BSP-targeting short hairpin RNA. The stable A549 BSP-shRNA clone used in this study was previously established and validated for BSP knockdown at the protein level [31]. Adherent cells were incubated overnight with 0.5 mL of the lentiviral preparation under standard culture conditions. The suspension containing the virus was then substituted with fresh complete medium, and transduced cells were selected using 1 μg/mL puromycin.

### 4.3. Assessment of Cell Viability

Cell viability was measured with an MTT-based assay in accordance with previously documented methodologies [58,59]. Cells were seeded in 96-well plates at a density of 5 × 10^3^ cells per well and subjected to the specified concentrations of BSP for durations of 24 or 48 h. Subsequent to treatment, the cells were washed with phosphate-buffered saline and incubated with 0.5 mg/mL MTT for one hour at 37 °C. The obtained formazan crystals were solubilized in DMSO, and absorbance was quantified at 570 nm utilizing a microplate reader.

### 4.4. Colony Formation

To assess cell proliferation, Cells were inoculated in 6-well plates at a concentration of 1 × 10^3^ cells per well in 2 mL of culture medium. Following a 24 h adhesion phase, the cells were subjected to incubation with the specified concentrations of BSP for an additional 24 h. The treatment media were then as desired; the cultures were washed once with PBS, and new complete medium was applied. Colonies were allowed to mature for 7 days prior to fixation using 3.7% formaldehyde and subsequent staining with 0.5% crystal violet for 15 min [60].

### 4.5. Wound Healing

After the achievement of cell monolayer confluence, mitomycin C was applied at a working concentration of 10 μg/mL for the duration of 2 h to suppress cellular growth throughout the experiment. A disinfected 1 mL pipette tip was subsequently employed to establish a linear cell-free interval across each monolayer. The cultures were thereafter subjected to BSP at concentrations of 0, 50, 100, or 200 ng/mL. Photographs of the injury site were captured at 24 h intervals using the Sage Vision imaging apparatus (Sage Vision Co., Ltd., New Taipei City, Taiwan). The remaining cell-free region was quantified with ImageJ (version 1.54g; National Institutes of Health, Bethesda, MD, USA) and represented in relation to the initial wound area documented at the start of the experiment.

### 4.6. Western Blot Analysis

Cellular proteins were collected using SDS lysis buffer mixed with a protease inhibitor cocktail. Protein concentrations were measured with the Pierce™ BCA Protein Assay Kit (Thermo Fisher Scientific, Waltham, MA, USA). Identical quantities of protein were analyzed using SDS–PAGE and then applied to membranes that bind proteins. The membranes were probed with primary antibodies targeting ADAM9, BSP, phosphorylated FAK, total FAK, or β-actin, followed by the corresponding HRP-conjugated secondary antibodies. β-Actin was used as the loading control for Western blot analysis. Immunoreactive signals were detected utilizing the iBright™ CL1500 Imaging System (Thermo Fisher Scientific, Waltham, MA, USA).

### 4.7. Real-Time Quantitative PCR (RT-qPCR)

Cellular mRNA was purified using TRIzol reagent (MDBio Inc., Taipei, Taiwan). An aliquot of 1 μg total RNA from each sample was used for first-strand synthesis of cDNA using the M-MLV reverse transcription kit, following the supplier’s recommendations. The resulting cDNA was subsequently subjected to quantitative amplification using a StepOnePlus™ Real-Time PCR System (Applied Biosystems, Waltham, MA 02451, USA).

### 4.8. Luciferase Activity

Cells were transfected with a luciferase reporter plasmid carrying either the wild-type ADAM9 3′-UTR miR-373-3p recognition sequence or a mutant sequence, together with a miR-373-3p mimic or its negative control. Nucleic acid delivery was performed using Lipofectamine™ 2000. Reporter activity was subsequently quantified with the Dual-Luciferase^®^ Reporter Assay System (Promega) in accordance with the supplier’s protocol.

### 4.9. Transfection

A549 and CL1-5 cells were transiently delivered with a miR-373-3p mimic or its negative control, or with ADAM9-specific siRNA or the corresponding control siRNA. Lipofectamine™ 2000 (Invitrogen, Carlsbad, CA, USA) was used as the transfection agent following the guidelines provided by the manufacturer.

### 4.10. Immunohistochemistry (IHC)

Human lung cancer tissue samples were gathered after the acquisition of written informed consent from each patient and with the endorsement of the Ethics Review Board of China Medical University Hospital (approval no. CMUH114-REC3-165; approval date: 26 September 2025). Paraffin-embedded sections underwent deparaffinization in xylene and were then rehydrated using a graded series of ethanol and exposed to the specified primary antibodies overnight at 4 °C. Immunohistochemical staining was subsequently completed according to previously established procedures. In brief, for IHC quantification, six lung cancer specimens were analyzed, including three non-metastatic and three metastatic cases. For each specimen, one tumor field was evaluated at 20× magnification. Staining intensity was scored on a scale from 0 to 5, with higher scores indicating stronger staining, and the mean score across the analyzed fields was used as the value for each patient. IHC evaluation was performed by an investigator who was blinded to the clinical grouping and patient information.

### 4.11. Databases Analysis

The Gene Expression Profiling Interactive Analysis 2 (GEPIA2) platform was used to evaluate the prognostic significance of SIBLING family members. The Cancer Genome Atlas lung adenocarcinoma cohort (TCGA-LUAD) dataset was used to analyze the expression patterns of ADAM family genes in lung adenocarcinoma. The TIMER2.0 database was used to further assess the correlations between BSP expression and specific ADAM genes.

### 4.12. Animal Models

To assess the role of BSP in lung cancer metastasis in an animal model, 6-week-old male BALB/c nude mice (approximately 22 g each) were obtained from BioLASCO Taiwan Co., Ltd. (Taipei, Taiwan). The animals were randomly assigned to two experimental groups, containing three mice in each cohort (sample size was established based on similar prior research [61]). Each mouse was administered an intra-arterial injection into the caudal artery including 1 × 10^6^ A549 cells or A549 cells exhibiting stable BSP knockdown facilitated by short hairpin RNA (A549 BSP shRNA cells). The metastatic progression was assessed eight weeks after tumor-cell injection using a Xenogen IVIS Imaging System 200 (PerkinElmer, Waltham, MA, USA). During bioluminescence imaging, the mice were subjected to anesthesia with 1.5 percent isoflurane. Throughout the study, the animals were maintained in specialized pathogen-free environments, given unrestricted access to food and drink, and kept under a 12 h light and 12 h dark cycle. Following the conclusive imaging evaluation, the mice were humanely terminated by CO_2_ inhalation. Lung tissues and hindlimb bones were then harvested and analyzed using hematoxylin and eosin (H&E) staining and IHC examination. All experimental techniques involving animals were executed in compliance with institutional guidelines and obtained clearance from the Institutional Animal Care and Use Committee at China Medical University (approval no. CMUH-IACUC-SN2025-114; approval date: 13 May 2025). Investigators were oblivious to the allocation of treatments throughout the assessment of results. No creatures or data items were excluded from the evaluation.

### 4.13. Statistical Analysis

Data are presented as the mean ± standard deviation (SD). All reported replicates represent independent biological replicates. Comparisons between two independent groups were performed using an unpaired two-tailed Student’s *t*-test. For experiments involving three or more groups, one-way analysis of variance (ANOVA) was followed by Dunnett’s multiple-comparisons test when multiple groups were compared with a single control, or Tukey’s multiple-comparisons test when multiple pairwise comparisons were required. The number of biological replicates (n) and the specific statistical comparisons are indicated in the corresponding figure legends. Statistical analyses were performed using GraphPad Prism version 8.0 (GraphPad Software, La Jolla, CA, USA). A *p* value < 0.05 was considered statistically significant.

## 5. Conclusions

This study identifies BSP as an upstream regulator of ADAM9 in lung cancer. BSP activates FAK signaling and modulates the hsa_circ_0084050/miR-373-3p axis, thereby increasing ADAM9 expression and promoting cancer cell proliferation, migration, and metastatic dissemination. BSP depletion was also associated with reduced metastatic burden in an exploratory mouse model. However, given the limited animal sample size and the use of male mice only, these in vivo findings should be interpreted cautiously and require confirmation in larger, adequately powered studies including both sexes.

## Data Availability

The original contributions presented in this study are included in the article/Supplementary Materials. Further inquiries can be directed to the corresponding authors.

## Supplementary Materials

The following supporting information can be downloaded at: https://www.mdpi.com/article/10.3390/ijms27177840/s1.
