# Supplementary material for: The hsa_circ_0084050/miR-373-3p Axis Controls Bone Sialoprotein-Induced ADAM9 Upregulation to Drive Lung Cancer Progression and Metastasis
Source: Int J Mol Sci. 2026 Sep 1;27(17):7840. doi: 10.3390/ijms27177840 (PMC13565926; doi:10.3390/ijms27177840)
Supplement: Supplementary file 1 [file ijms-27-07840-s001.zip › ijms-4476671-supplementary.pdf]

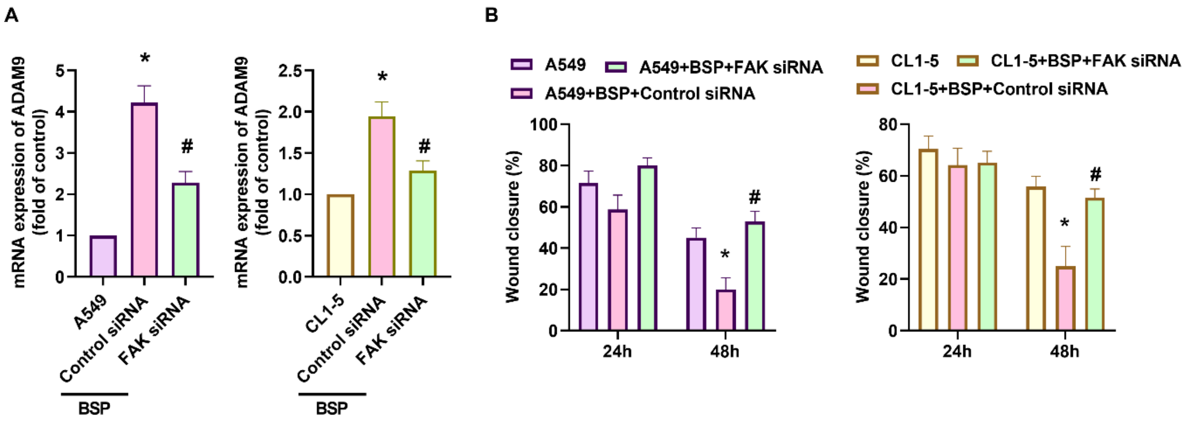

Figure S1. FAK silencing attenuates BSP-induced ADAM9 expression and cell migration in lung cancer cells. (A) ADAM9 mRNA expression in A549 and CL1-5 cells following BSP treatment in the presence of control siRNA or FAK siRNA, determined by RT-qPCR. (B) Effects of FAK silencing on BSP-regulated cell migration in A549 and CL1-5 cells, evaluated by wound-healing assay at 24 and 48 h. Data are presented as mean ± SD from three independent biological experiments (n = 3). Statistical significance was determined by one-way ANOVA followed by Tukey's multiple-comparisons test, with analyses performed separately for each cell line and time point. $p < 0.05$ versus the corresponding untreated control; #$p < 0.05$ versus the BSP + control siRNA group.
